# Intelligent Prediction Platform for Sepsis Risk Based on Real-Time Dynamic Temporal Features: Design Study

**DOI:** 10.2196/74940

**Published:** 2025-05-30

**Authors:** Mingwei Zhang, Ming Zhong, Yunzhang Cheng, Tianyi Zhang

**Affiliations:** 1 School of Health Sciences and Engineering University of Shanghai for Science and Technology Shanghai China; 2 Department of Critical Care Medicine Zhongshan Hospital Affiliated to Fudan University Shanghai China; 3 Lin-gang Medical Device Innovation Center Shanghai China

**Keywords:** sepsis, real-time dynamic warnings, machine learning, interpretability, web-based platform

## Abstract

**Background:**

The development of sepsis in the intensive care unit (ICU) is rapid, the golden rescue time is short, and the effective way to reduce mortality is rapid diagnosis and early warning. Therefore, real-time prediction models play a key role in the clinical diagnosis and management of sepsis. However, the existing sepsis prediction models based on artificial intelligence still have limitations, such as poor real-time performance and insufficient interpretation.

**Objective:**

Our objective is to develop a real-time sepsis prediction model that integrates high timeliness and clinical interpretability. The model is designed to dynamically predict the risk of sepsis in ICU patients and establish a practical, tailored sepsis prediction platform.

**Methods:**

Within a retrospective analysis framework, the model comprises a real-time prediction module and an interpretability module. The real-time prediction module leverages 3-hour dynamic temporal features derived from 8 noninvasive, real-time physiological indicators: heart rate, respiratory rate, blood oxygen saturation, mean arterial pressure, systolic blood pressure, diastolic blood pressure, body temperature, and blood glucose. Three linear parameters (mean, SD, and endpoint value) were calculated to construct the prediction model using multiple ML algorithms. The interpretability module uses the TreeSHAP (Tree-Based Shapley Additive Explanations) method to enhance model transparency through both individual prediction and global explanations. Further, it added a link between the output interpretation of the explainable artificial intelligence method and its potential physiological or pathophysiological significance, including the relationship among the output interpretation and the patient’s hemodynamics, thermoregulatory response, and the balance between oxygen delivery and oxygen consumption. Finally, a web-based platform was developed to integrate prediction and interpretability functions.

**Results:**

The sepsis prediction model demonstrated robust performance in the test cohort (224 patients), achieving an accuracy of 0.7 (95% CI 0.68-0.71), precision of 0.69 (95% CI 0.68-0.71), *F*_1_-score of 0.69 (95% CI 0.67-0.70), and area under the receiver operating characteristic curve of 0.76 (95% CI 0.74-0.77). The TreeSHAP method effectively visualized feature contributions, enabling clinicians to interpret the model’s prediction logic and identify anomalies. The link between the output interpretation of the model and its potential physiological or pathophysiological significance improved the interpretability and credibility of the explainable artificial intelligence method. The web-based platform significantly enhanced clinical utility by providing real-time risk assessment, statistical summaries, trend analysis, and actionable insights.

**Conclusions:**

This platform provides real-time dynamic warnings for sepsis risk in critically ill ICU patients, supporting timely clinical decision-making.

## Introduction

Sepsis, a life-threatening clinical syndrome triggered by infection, continues to impose a significant global burden due to its persistently high incidence and mortality rates. In the United States, approximately 750,000 individuals are diagnosed with sepsis annually, with a mortality rate exceeding 30%; Europe reports 150,000 sepsis-related deaths yearly [1,2]. The economic impact is staggering, with annual US health care costs exceeding US $20.3 billion [3]. Patients with sepsis experience hospital stays twice as long as those with other conditions, and the incidence of severe sepsis continues to rise by 13% annually [4]. Early detection is critical to reducing mortality; yet, current diagnostic methods lack accuracy and real-time capability [5]. The multifactorial characteristics of sepsis increase the difficulty of early diagnosis, and the specificity of its diagnostic indicators is relatively low, which is prone to cause misdiagnosis [6]. Timely initiation of protocolized treatment bundles significantly improves survival [7], with evidence suggesting that a 1-hour resuscitation bundle may become the cornerstone of septic shock management [8]. However, sepsis pathophysiology is complex, microbiological confirmation is time-consuming, and existing scoring systems (eg, Mortality in Emergency Department Sepsis or Sequential Organ Failure Assessment) have limitations [9]. Thus, establishing a real-time sepsis prediction system is imperative to reduce clinical mortality.

Current research has leveraged artificial intelligence (AI) to develop sepsis prediction models using dynamic physiological and laboratory data. Machine learning has gained traction in critical care for disease diagnosis [10], outcome prediction [11-13], and clinical decision support [14]. Recent AI models for sepsis prediction outperform traditional methods [15-18], but reliance on non–real-time laboratory data and the “black-box” nature of AI models hinder clinical adoption [19,20]. These models often fall short in real-time performance and interpretability, limiting their clinical utility. Intensive care unit (ICU) clinicians require transparent, medically logical AI tools to preserve decision-making autonomy, aligning with evidence-based principles. Current sepsis prediction models also lack integration into practical platforms, further limiting clinical utility.

This study addresses these gaps by developing ML models based on dynamic features from real-time, noninvasive physiological indicators. We use local and global interpretability methods to enhance clinical trust and establish a web-based sepsis prediction platform.

## Methods

### Data Source

Data were extracted from the MIMIC-IV (Medical Information Mart for Intensive Care IV) database, developed by the MIT (Massachusetts Institute of Technology) Laboratory for Computational Physiology. This deidentified database includes clinical and waveform data from ICU and emergency department patients at Beth Israel Deaconess Medical Center (2008-2019) [21,22]. The physiological indicators were obtained from patient monitors. Blood oxygen saturation (SpO_2_), heart rate (HR), and respiratory rate (RES) were collected 1-10 times per hour. Blood glucose (GLU) and body temperature (TEM) were measured once every 1 to 5 hours, depending on the patient’s condition. Other indicators were collected hourly.

### Study Cohort and Variable Selection

Patients with sepsis were identified per the 2018 Chinese Guidelines for Sepsis or Septic Shock Management (positive blood culture + antibiotic use + Sequential Organ Failure Assessment score ≥2). A control cohort included ICU patients without sepsis. Among 1118 patients (550 with sepsis and 568 controls), 8 real-time physiological indicators were selected: HR, systolic blood pressure (SP), diastolic blood pressure (DP), mean arterial pressure (MP), RES, TEM, SpO_2_, and GLU. Stratified sampling divided the cohort into training (n=894) and test (n=224) groups. Baseline characteristics showed no significant differences (Table 1).

**Table 1:** *t* test analysis of characteristics between training and test groups^a^.

Parameter	Training (n=894), median (IQR)	Test (n=224), median (IQR)	*P* value
Heart rate (bpm)	86.1 (30.5-157)	86.5 (43-137.5)	.81
Systolic BP^b^ (mm Hg)	119.6 (48-191)	115.5 (36.8-187)	.08
Diastolic BP (mm Hg)	61.5 (10-116)	61.9 (19-121)	.67
Mean arterial pressure (mm Hg)	77.8 (24.3-149)	75.7 (8-127)	.08
Respiratory rate (bpm)	19.8 (5-44)	19.6 (6-52)	.60
Temperature (°C)	36.8 (31.7-40.4)	36.8 (33.1-39.9)	.74
SpO_2_^c^ (%)	96 (28.4-100)	95.6 (23-100)	.42
Blood glucose (mg/dL)	140.6 (42-309)	139.3 (63-326)	.75

^a^*P*<.05 indicates statistical significance.

^b^BP: blood pressure.

^c^SpO_2_: blood oxygen saturation.

### Data Preprocessing

First, the data underwent outlier removal. Since part of the MIMIC-IV data was manually entered by health care providers, potential input errors or anomalies were addressed. Clinical knowledge was applied to define valid physiological ranges and filter absolute outliers (for instance, TEM: 20 °C-50 °C; SpO_2_: 21%-100%; HR: 0-300 bpm). Data points outside these ranges were deemed invalid and removed. Next, missing data imputation was performed to address gaps in the cleaned dataset. A hybrid approach combining multiple interpolation methods was applied:

1. Mean imputation: Replacing missing values with the overall mean of the feature.

2. Class-specific mean imputation: Using mean values from subgroups (eg, sepsis vs nonsepsis cohorts).

3. Linear interpolation: Filling gaps using linear trends between adjacent valid data points.

4. Forward-fill imputation: Propagating the last valid observation forward.

**Figure 1:**
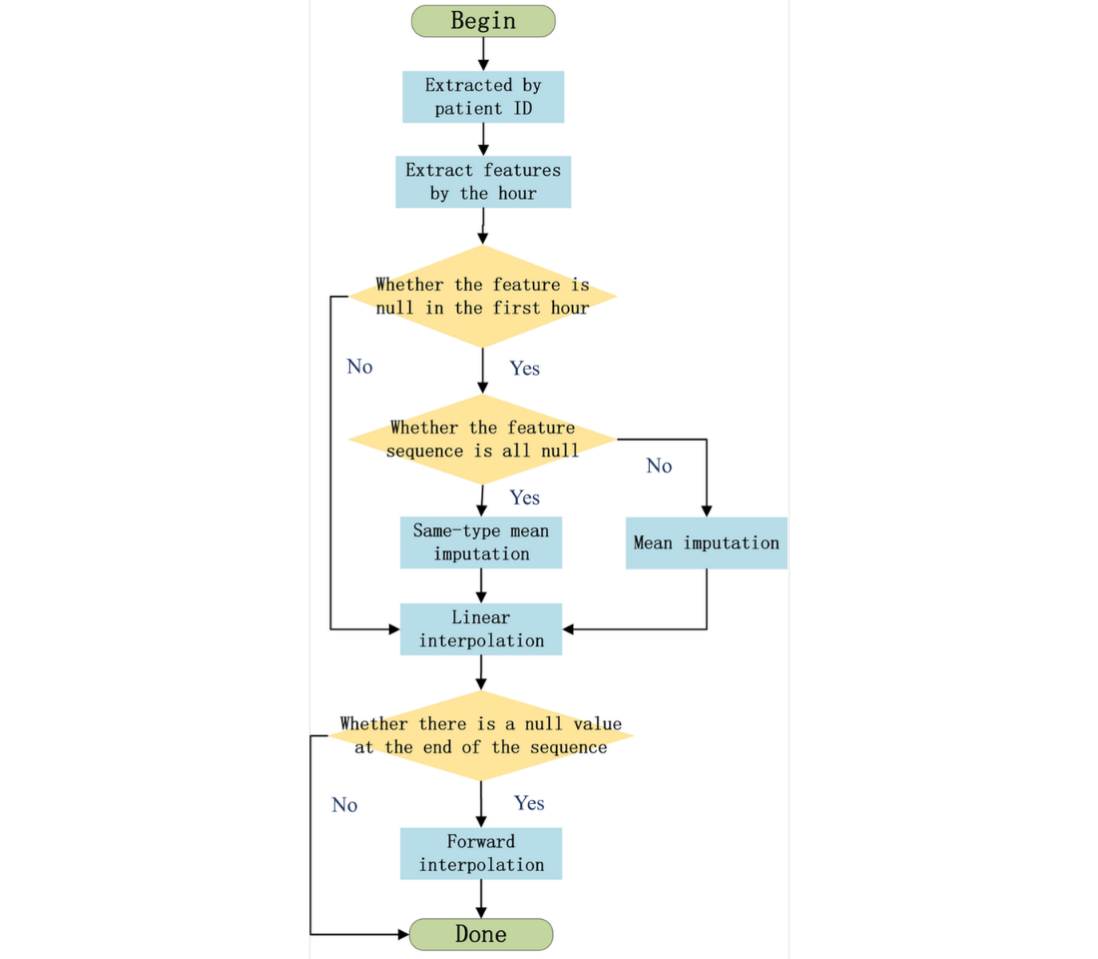
The entire preprocessing workflow.

The entire preprocessing workflow is illustrated in Figure 1.

To minimize reliance on long-term temporal dependencies, the prediction model was designed to operate on a 3-hour sliding window of real-time physiological data. This allows the model to initiate sepsis risk prediction immediately after 3 hours of monitoring. A 3-hour window allows sufficient time for sign monitoring and subsequent sepsis prediction, providing doctors with ample time for intervention. From the 3-hour time series of each physiological indicator, 3 linear parameters were computed: mean value over the 3-hour window, fluctuation coefficient (SD within the window), and endpoint value (the last recorded value in the window).

This generated a 24-dimensional feature vector for each patient, structured as follows:

- Endpoint values: HR, SP, DP, MP, RES, TEM, SpO_2_, and GLU.
- Mean values: mean-HR, mean-SP, mean-DP, mean-MP, mean-RES, mean-TEM, mean-SpO_2_, and mean-GLU.
- Fluctuation coefficients (SDs): var-HR (variation in heart rate), var-SP (variation in systolic blood pressure), var-DP, var-MP (variation in mean arterial pressure), var-RES (variation in respiratory rate), var-TEM (variation in body temperature), var-SpO_2_ (variation in blood oxygen saturation), and var-GLU (variation in blood glucose).

All features were standardized (*z* score normalization) to ensure consistent scaling before model training. This preprocessing pipeline ensures robust, clinically meaningful inputs for the subsequent machine learning workflow.

### Sepsis Prediction Model

We developed and evaluated the sepsis prediction model for actual ICU sign monitoring to achieve a model with greater generality in a broader clinical setting. Critically ill patients in the ICU have different needs for GLU monitoring frequency. The incidence of glucose metabolism disorders in critically ill patients is high, and the incidence increases in turn in sepsis, severe sepsis and septic shock [23], which not only reflects the abnormal secretion of hormones and the severity of the disease, but is also closely related to the increase of mortality and complications [24]. High or low GLU is one of the causes of organ dysfunction. Therefore, it is necessary to increase the frequency of GLU monitoring in the face of the clinical background of insufficient energy intake, high catabolism, impaired GLU regulation mechanism, or the implementation of insulin treatment. At the same time, it is also necessary to increase the frequency of GLU monitoring in critically ill patients with diabetes or hypoglycemia. Whereas in most other ICU patients, GLU is usually measured only once or twice a day.

Therefore, to improve the generalization of sepsis prediction models, we developed and evaluated 2 sepsis prediction models, one based on high-frequency glucose monitoring and the other based on routine vital signs monitoring without glucose. By combining the 2 models, a model with greater generality in a wider range of clinical settings was achieved.

This study used 6 machine learning algorithms—support vector machine, random forest, ExtraTrees, XGBoost (Extreme Gradient Boosting), AdaBoost, and Logistic—to construct real-time sepsis prediction models. Model performance was evaluated and compared using metrics including accuracy, precision, recall, *F*_1_-score, and area under the receiver operating characteristic curve (AUROC).

### Model Interpretation

The TreeSHAP (Tree-Based Shapley Additive Explanations) method was used to provide both local (individual prediction) and global explanations for the sepsis prediction model. TreeSHAP, grounded in cooperative game theory, constructs an additive explanation model that treats all features as “contributors” to the prediction outcome. For each sample, TreeSHAP generates a Shapley value for each feature, quantifying its marginal contribution to the model’s decision. For the sepsis prediction model with 24 features, let the original model be f, trained using AdaBoost. The explanation model g in SHAP (Shapley Additive Explanations) is defined as:




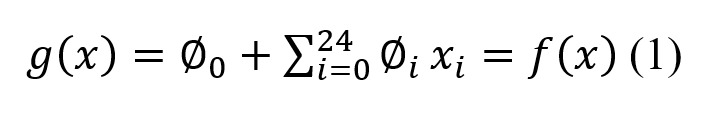




x=(x_1_,x_2_…x_24_) represents the feature vector of a single patient. f(x) is the prediction from the original model. g(x) is the prediction from the explanation model. ø_i_ is the Shapley value for the ith feature.




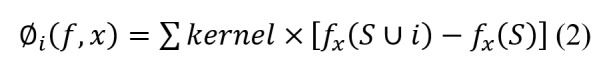







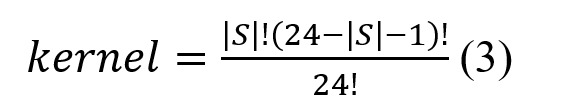




S is a subset of {1,2,3…24}, with 2^24-1^ possible combinations. |S| is the number of features in subset S. f_x_ (S ∪ i) and f_x_(S) are model predictions with and without feature i, respectively. Global interpretation aggregates local explanations by averaging the absolute Shapley values across all samples.

### Ethical Considerations

Our study was conducted in accordance with the guidelines of the Helsinki Declaration. The Review Committee of the Massachusetts Institute of Technology and Beth Israel Deaconess Medical Center approved access to the MIMIC-IV database. Authors fulfilled the database access request. All these data were deidentified; therefore, the study was exempt from ethical approval and informed consent requirements.

## Results

### Model Prediction Performance

**Figure 2:**
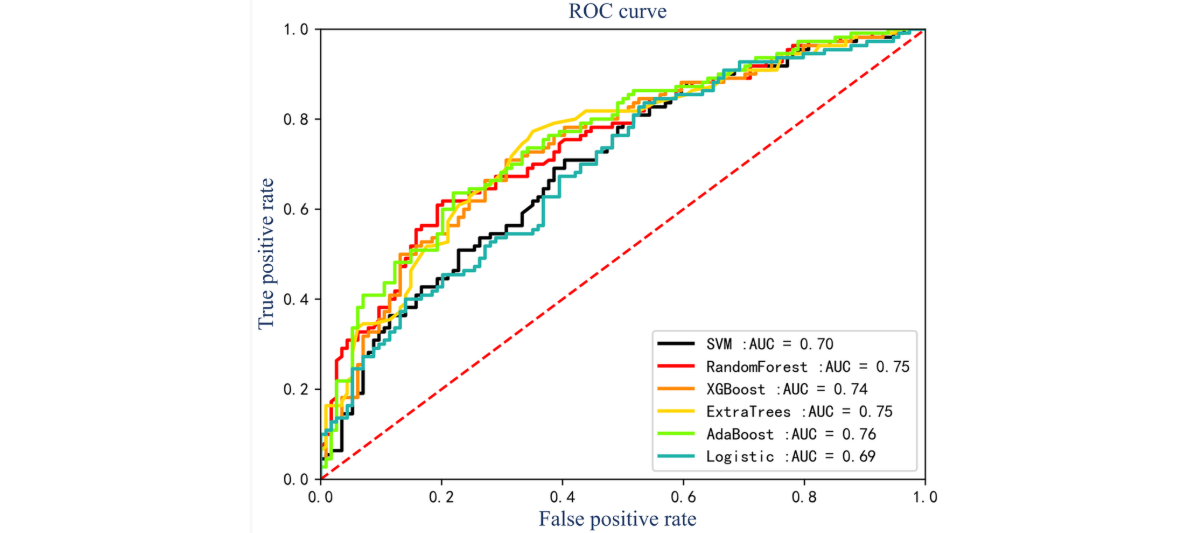
Receiver operating characteristic curves of machine learning models based on high-frequency glucose monitoring. AUC: area under the curve; ROC: receiver operating characteristic; SVM: support vector machine; XGBoost: Extreme Gradient Boosting.

**Figure 3:**
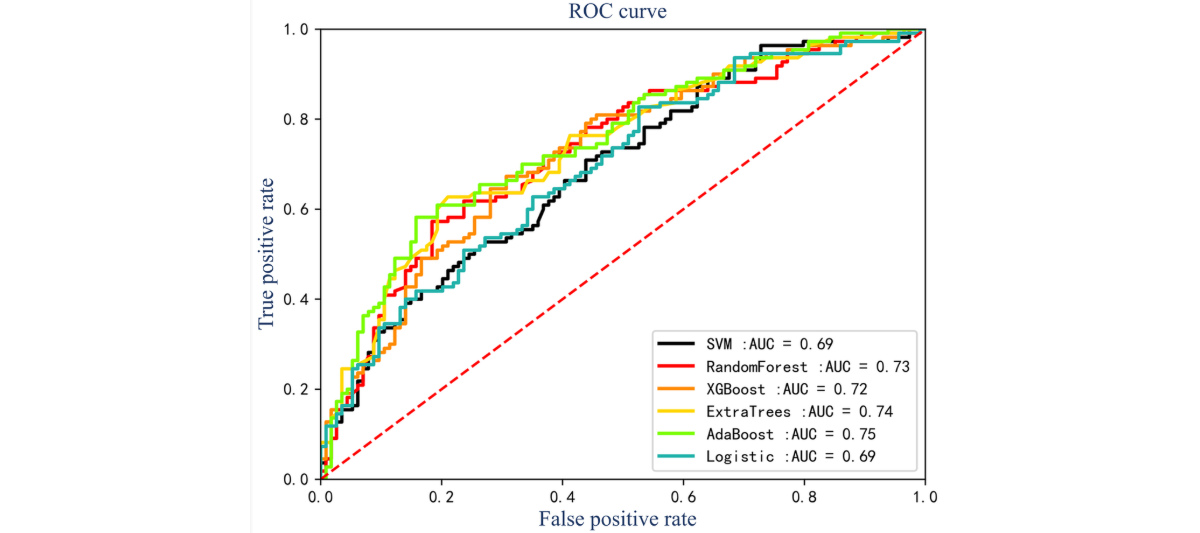
Receiver operating characteristic curves of machine learning models based on routine vital signs monitoring without glucose. AUC: area under the curve; ROC: receiver operating characteristic; SVM: support vector machine; XGBoost: Extreme Gradient Boosting.

The performances of the sepsis prediction models based on high-frequency glucose monitoring are summarized in Figure 2 and Table 2. Among all models, AdaBoost achieved the highest accuracy of 0.70 (95% CI 0.68-0.71), precision of 0.69 (95% CI 0.68-0.71), *F*_1_-score of 0.69 (95% CI 0.67-0.70), and AUROC of 0.76 (95% CI 0.74-0.77), demonstrating superior real-time prediction capability. The performances of the sepsis prediction models based on routine vital signs monitoring without glucose are summarized in Figure 3 and Table 3. Among all models, AdaBoost achieved the highest accuracy of 0.67 (95% CI 0.66-0.69), precision of 0.67 (95% CI 0.65-0.68), and AUROC of 0.75 (95% CI 0.74-0.77). The experimental results show that the sepsis prediction model based on high-frequency monitoring of GLU has better prediction performance, and the sepsis prediction model based on routine vital signs monitoring has a wider application scenario.

**Table 2:** Models’ performance comparison of machine learning models based on high-frequency glucose monitoring.

Models	Accuracy, median (IQR)	Precision, median (IQR)	Recall, median (IQR)	*F*_1_-score, median (IQR)	AUROC^a^, median (IQR)
SVM^b^	0.64 (0.61-0.66)	0.64 (0.62-0.67)	0.6 (0.59-0.61)	0.62 (0.6-0.65)	0.7 (0.69-0.72)
Random forest	0.68 (0.65-0.7)	0.65 (0.62-0.67)	0.7 (0.68-0.73)	0.67 (0.65-0.69)	0.75 (0.74-0.77)
ExtraTrees	0.69 (0.67-0.71)	0.69 (0.67-0.71)	0.66 (0.65-0.68)	0.67 (0.65-0.7)	0.75 (0.72-0.78)
XGBoost^c^	0.7 (0.68-0.72)	0.68 (0.68-0.69)	0.71 (0.7-0.72)	0.69 (0.68-0.69)	0.74 (0.73-0.75)
AdaBoost	0.7 (0.68-0.71)	0.69 (0.68-0.71)	0.67 (0.66-0.69)	0.69 (0.67-0.7)	0.76 (0.74-0.77)
Logistic	0.63 (0.62-0.63)	0.62 (0.61-0.62)	0.62 (0.61-0.63)	0.62 (0.62-0.63)	0.69 (0.67-0.7)

^a^AUROC: area under the receiver operating characteristic curve.

^b^SVM: support vector machine.

^c^XGBoost: Extreme Gradient Boosting.

**Table 3:** Models’ performance comparison of machine learning models based on routine vital signs monitoring without glucose.

Models	Accuracy, median (IQR)	Precision, median (IQR)	Recall, median (IQR)	*F*_1_-score, median (IQR)	AUROC^a^, median (IQR)
SVM^b^	0.61 (0.59-0.64)	0.61 (0.59-0.63)	0.57 (0.55-0.6)	0.59 (0.57-0.61)	0.69 (0.67-0.71)
Random forest	0.66 (0.65-0.68)	0.65 (0.62-0.67)	0.68 (0.66-0.71)	0.66 (0.64-0.69)	0.73 (0.72-0.74)
ExtraTrees	0.67 (0.65-0.7)	0.65 (0.63-0.67)	0.66 (0.65-0.68)	0.66 (0.65-0.67)	0.74 (0.72-0.76)
XGBoost^c^	0.67 (0.66-0.69)	0.65 (0.63-0.67)	0.73 (0.71-0.74)	0.68 (0.66-0.7)	0.72 (0.7-0.73)
AdaBoost	0.67 (0.66-0.69)	0.67 (0.65-0.68)	0.66 (0.64-0.68)	0.66 (0.65-0.67)	0.75 (0.74-0.77)
Logistic	0.64 (0.63-0.65)	0.63 (0.62-0.64)	0.63 (0.61-0.64)	0.63 (0.62-0.63)	0.69 (0.67-0.7)

^a^AUROC: area under the receiver operating characteristic curve.

^b^SVM: support vector machine.

^c^XGBoost: Extreme Gradient Boosting.

### Interpretability Analysis Using TreeSHAP

#### Individual Prediction Explanation

**Figure 4:**
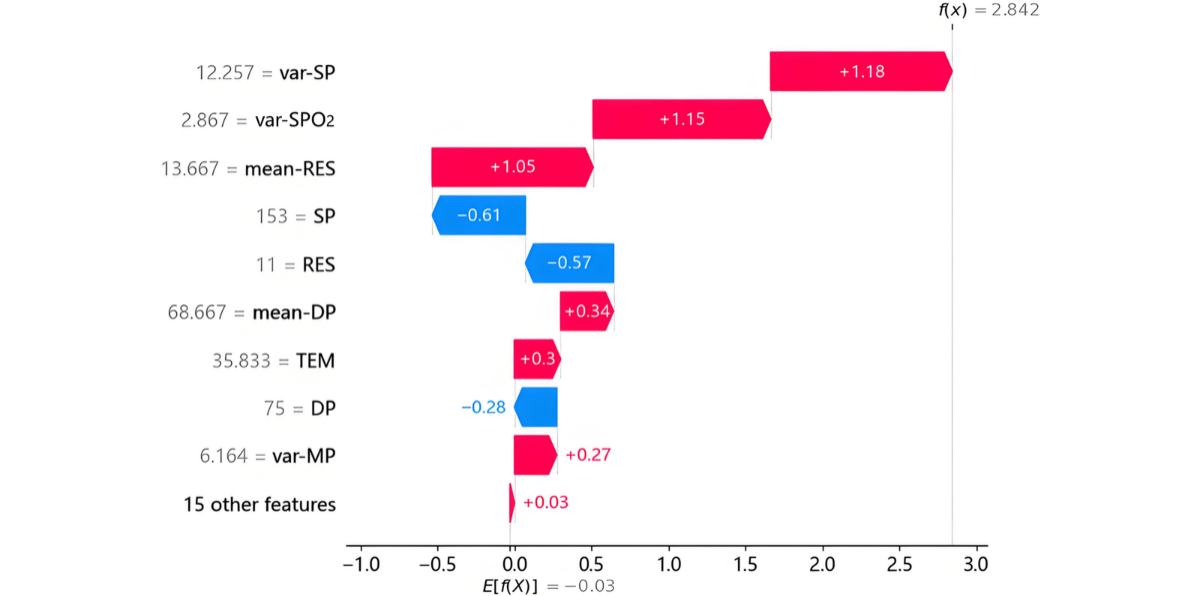
Individual prediction explanation. DP: diastolic blood pressure; MP: mean arterial pressure; RES: respiratory rate; SP: systolic blood pressure; SpO_2_: blood oxygen saturation; TEM: body temperature.

The TreeSHAP method was used to generate local explanations for individual cases to elucidate the contribution of dynamic features to model predictions. Figure 4 illustrates the interpretability analysis for a patient predicted as having sepsis, where features are color-coded to reflect their impact: red denotes a positive contribution (increasing sepsis risk) and blue indicates a negative contribution (reducing sepsis risk). The baseline value E[f(x)], representing the model’s average prediction across the dataset, serves as the reference point. Key findings include: SP fluctuation coefficient (var-SP=12.257) exerted the strongest positive influence (+1.18), followed by SpO_2_ fluctuation (var-SpO_2_=2.867), mean RES (mean-RES=3.667), mean DP (mean-DP=68.667), TEM (TEM=35.833), and MP fluctuation (var-MP=6.164). Negative contributors included SP (SP=15), RES (RES=11), and DP (DP=75). These results basically align with clinical intuition, where elevated variability in hemodynamic parameters (eg, var-SP or var-MP), hypothermia, and reduced RES correlate with sepsis pathophysiology. The interpretability framework enables clinicians to validate model logic against established diagnostic criteria and identify anomalous predictions.

#### Global Interpretation

**Figure 5:**
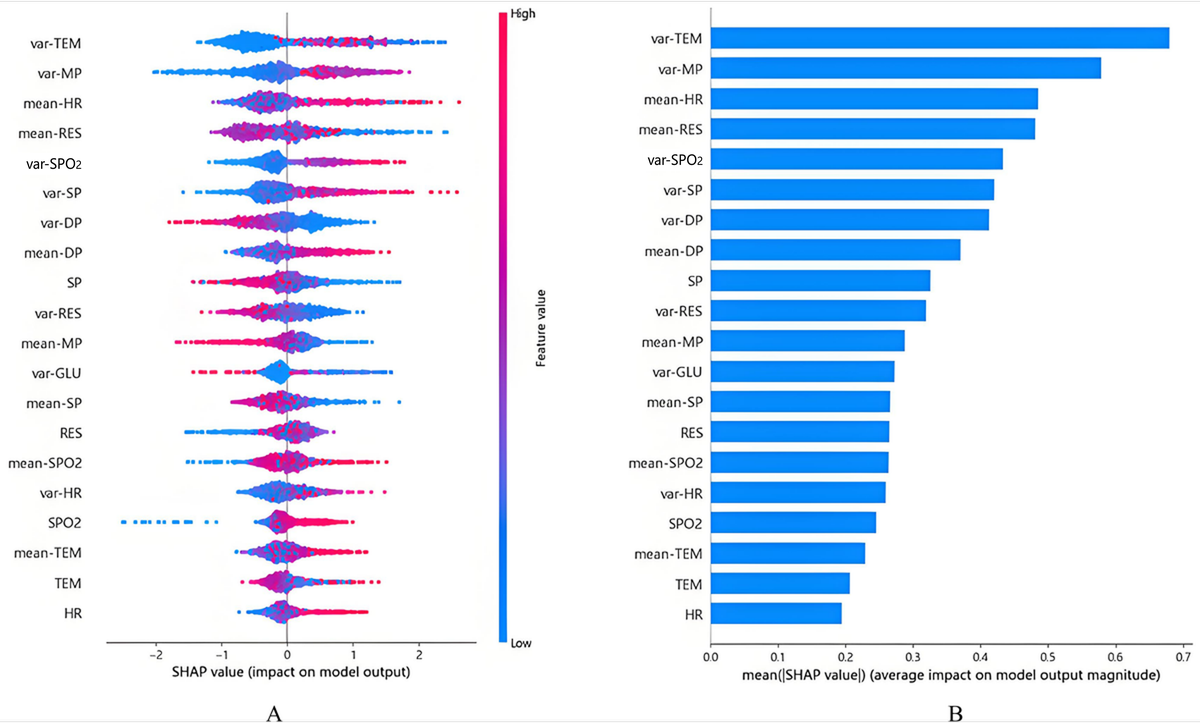
Global interpretation of the model and feature importance based on mean Shapley values. DP: diastolic blood pressure; GLU: blood glucose; HR: heart rate; MP: mean arterial pressure; RES: respiratory rate; SHAP: Shapley Additive Explanations; SP: systolic blood pressure; SpO_2_: blood oxygen saturation; TEM: body temperature.

Global interpretability analysis aggregated Shapley values across all samples to reveal the model’s overarching decision logic and feature importance rankings (Figure 5). As illustrated in Figure 5A, the model demonstrated associations between decreased SP and elevated sepsis risk (row 9), increased SP fluctuation (var-SP) and elevated sepsis risk (row 6), and increased MP fluctuation (var-MP) with elevated sepsis risk (row 2). These interpretations align closely with existing clinical evidence and provide clinically meaningful insights that are of particular interest to clinicians. For instance, increased var-SP is more likely to elevate the probability of sepsis compared to increased var-MP. The Surviving Sepsis Campaign 2021 guidelines emphasize the importance of SP in the diagnostic criteria for sepsis and recommend using SP<90 mm Hg after adequate fluid resuscitation as one of the indicators for evaluating septic shock [25]. Additionally, studies have shown that blood pressure variability in sepsis patients correlates with disease severity [26].

The connection between the parasympathetic nervous system and inflammation suggests interdependence between autonomic nervous system function and inflammatory responses [27]. Furthermore, physiological systems exhibit nonlinear patterns of complexity, including fractal self-similarity across time scales [28]. Previous research has documented an association between the complexity of autonomic nervous system control and hemodynamic instability, indicating that such complexity may serve as a potential window into understanding hemodynamic confusion. However, the relevance of these measures of complexity in sepsis remains unclear [26].

High temperature fluctuations (var-TEM) were strongly associated with increased sepsis probability, while stable temperatures reduced the risk (row 1). Notably, extreme TEMs, whether hyperthermia or hypothermia, were linked to elevated risks, whereas normal-range temperatures exhibited protective effects (row next-to-last). This interpretation of the model aligns with the clinical presentation of sepsis.

Both hypothermia and hyperthermia are generally associated with elevated lactate levels, and patients with severe sepsis often develop either hypothermia or, more commonly, a febrile response. Hypothermia in some patients with sepsis is well-documented and forms part of the definition of systemic inflammatory response syndrome [29]. There is variable thermoregulatory response in sepsis, and the definition of systemic inflammatory response syndrome in sepsis-1 includes both fever and hypothermia. The impact of thermoregulatory response on sepsis prognosis remains controversial. Studies have shown that hypothermia or fever can have either protective or detrimental effects in animal models of severe infection or inflammation [30]. Fever is a physiological response to infection that inhibits bacterial growth, prevents fungal proliferation, and enhances immune cell activity against pathogens [31]. Pathogens detected in blood cultures and elevated procalcitonin levels, both associated with high fever, indicate robust immune resistance to pathogen challenges [32].

The model revealed an association between increased variability in oxygen saturation (var-SpO_2_) and elevated sepsis risk (row 5). This finding underscores the necessity of maintaining SpO_2_ levels within a reasonable range for ICU patients during hospitalization, which is consistent with existing clinical evidence. Studies have demonstrated a U-shaped relationship between SpO_2_ levels and in-hospital all-cause mortality in patients with sepsis, where both hyperoxia and hypoxia are associated with increased mortality risk. The optimal SpO_2_ range is determined to be 0.96-0.98 [33]. Under normal physiological conditions, oxygen supply and consumption remain relatively stable, with SpO_2_ fluctuating within a normal range without extreme variation. During shock, however, the imbalance between oxygen supply and consumption leads to deviations in SpO_2_ values, resulting in increased var-SpO_2_. A multicenter observational study involving over 600 patients with sepsis confirmed that abnormal SpO_2_ levels (either abnormally high or low) were associated with increased mortality [34].

Figure 5B ranks feature mean absolute Shapley values, identifying the top 6 determinants of sepsis risk: temperature fluctuation (var-TEM), MP fluctuation (var-MP), mean HR (mean-HR), mean RES (mean-RES), SpO_2_ fluctuation (var-SpO_2_), and SP fluctuation (var-SP). These findings underscore the critical role of hemodynamic and thermoregulatory instability in sepsis onset, consistent with clinical biomarkers. The global analysis also highlights potential outliers, guiding model refinement and clinical vigilance.

#### Implementation of the Practical and Efficient Sepsis Risk Prediction Platform

**Figure 6:**
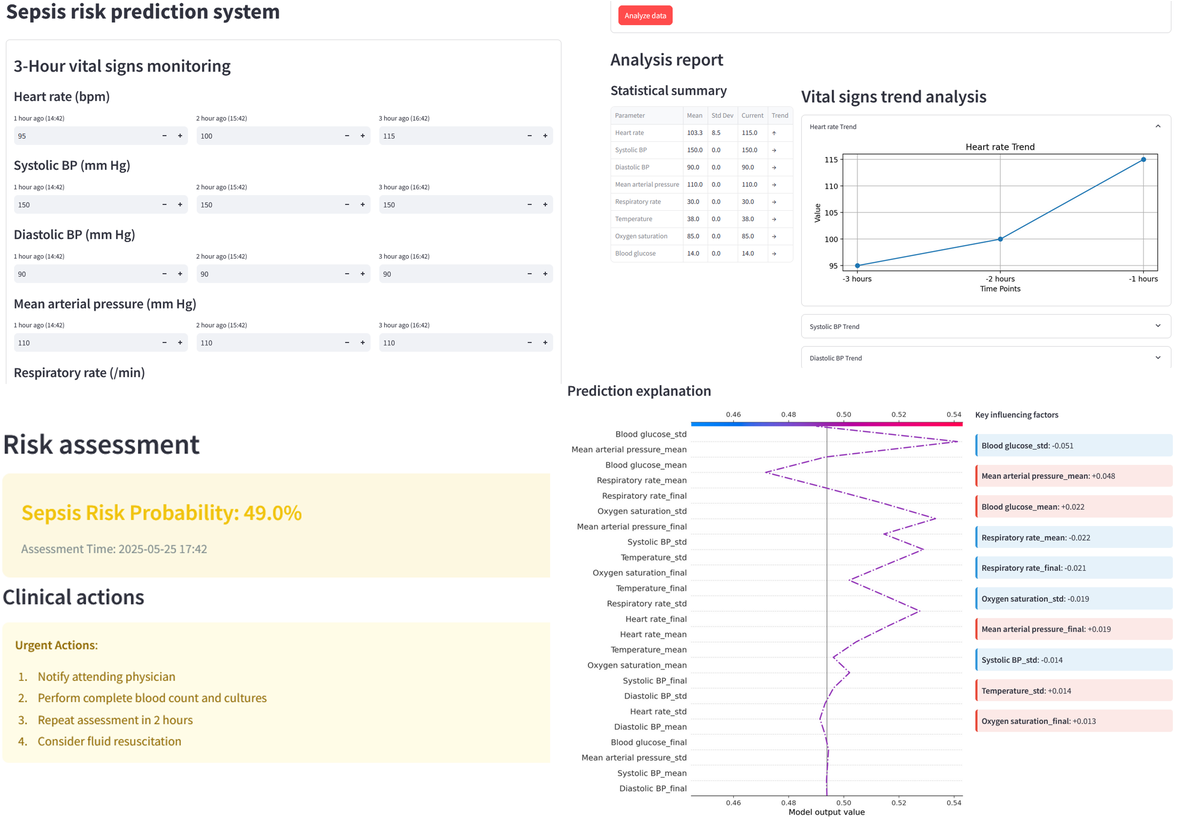
Practical and efficient sepsis risk prediction platform. BP: blood pressure.

We developed a practical and intelligent sepsis risk prediction platform using a web-based tool [35] (Figure 6). By monitoring 3-hour dynamic temporal features of ICU patients—including HR, SP, DP, MP, RES, TEM, SpO_2_, and GLU—the platform automatically generates a graphical analysis report comprising:

Statistical summary: quantitative analysis of dynamic temporal features (mean, SD, and endpoint values).

Vital signs trend analysis: visualization of physiological trends via time-series curves.

- Risk assessment: sepsis risk prediction using the trained model, based on calculated linear parameters.
- Clinical actions: automated recommendations (for reference only) tailored to risk stratification.
- Prediction explanation: visual display of key influencing factors using Shapley values to interpret model decisions.

This platform, built on real-time dynamic temporal features, enables personalized sepsis risk prediction for ICU patients, enhances clinical utility by integrating interpretable AI insights, and streamlines decision-making through intuitive data visualization and actionable outputs.

## Discussion

### Principal Findings

Our study developed a real-time sepsis prediction model that integrates high timeliness and clinical interpretability based on 3-hour dynamic temporal sequences of 8 rapidly accessible physiological indicators. The real-time sepsis prediction model demonstrated robust performance. The output interpretation of explainable artificial intelligence (XAI) enhanced model transparency through both individual prediction and global explanations, and it linked the potential physiological or pathophysiological significance, including the patient’s hemodynamics, thermoregulatory response, and the balance between oxygen delivery and oxygen consumption. Although current consensus emphasizes early intervention for sepsis management, the optimal predictors guiding intervention and markers of early sepsis severity remain unclear. In this study, we further elucidated the model’s output results, explored the potential relationships between relevant sign parameters and sepsis, and discussed their potential physiological or pathophysiological significance. This enhances the interpretability and credibility of the XAI method, supporting the model’s applicability in real-world clinical practice. Finally, the web-based platform significantly enhanced clinical utility by providing real-time risk assessment, statistical summaries, trend analysis, and actionable insights.

### Limitations

However, as a retrospective study, potential biases may exist in this study. Future efforts should prioritize multicenter validation and large-scale prospective studies to strengthen the robustness of these results. XAI holds immense promise in sepsis diagnosis and treatment, yet its development and clinical application face significant challenges [36]. Data quality remains a critical bottleneck. Heterogeneous hospital databases, inconsistent data collection and storage standards, and poor interoperability between health care information systems have led to fragmented “data silos,” hindering the application of large-scale clinical feature datasets. Additionally, the reliance on limited public or institution-specific databases in research settings restricts generalizability. In clinical practice, clinicians must integrate XAI predictions with laboratory findings (eg, lactate or procalcitonin), imaging results, and patient-specific symptoms to ensure accurate diagnosis and treatment planning.

### Conclusions

In ICU settings, real-time physiological indicators—such as HR, RES, and SpO_2_—are typically used to monitor symptom fluctuations rather than generate objective diagnostic reports. This limitation stems from the significant variability in individual physiological data, the complexity of multiparameter interactions, and the diverse clinical implications of these metrics. This study demonstrates that XAI can bridge this gap by synthesizing real-time physiological data into actionable insights. By analyzing dynamic trends and providing interpretable explanations, XAI uncovers the diagnostic potential of these data. Our model, focused on sepsis risk prediction, leverages real-time physiological features to generate predictions while emphasizing the interpretability of results. In the future, XAI systems could deliver intelligent diagnostic reports integrating disease prediction, anomaly detection, and causal analysis of abnormal indicators, empowering clinicians to navigate complex physiological data with precision.

## Abbreviations

AI: artificial intelligence
AUROC: area under the receiver operating characteristic curve
DP: diastolic blood pressure
GLU: blood glucose
HR: heart rate
ICU: intensive care unit
MIMIC-IV: Medical Information Mart for Intensive Care IV
MIT: Massachusetts Institute of Technology
MP: mean arterial pressure
RES: respiratory rate
SHAP: Shapley Additive Explanations
SP: systolic blood pressure
SpO_2_: blood oxygen saturation
TEM: body temperature
TreeSHAP: Tree-Based Shapley Additive Explanations
var-GLU: variation in blood glucose
var-HR: variation in heart rate
var-MP: variation in mean arterial pressure
var-RES: variation in respiratory rate
var-SP: variation in systolic blood pressure
var-SpO_2_: variation in blood oxygen saturation
var-TEM: variation in body temperature
XAI: explainable artificial intelligence
XGBoost: Extreme Gradient Boosting

## Data Availability

The data used in this study are publicly available from the Medical Information Mart for Intensive Care IV (MIMIC-IV) database. As a result of patient privacy concerns, the raw EHR (electronic health records) text data cannot be shared publicly but can be accessed through a compliant application to MIMIC-IV.

## References

[1] Angus D, Linde-Zwirble WT, Lidicker J, Clermont G, Carcillo J, Pinsky MR (2001). Epidemiology of severe sepsis in the United States: analysis of incidence, outcome, and associated costs of care. Crit Care Med.

[2] Stevenson EK, Rubenstein AR, Radin GT, Wiener RS, Walkey AJ (2014). Two decades of mortality trends among patients with severe sepsis: a comparative meta-analysis*. Crit Care Med.

[3] Moore B, Levit K, Elixhause A (2014). Costs for hospital stays in the United States, 2012: statistical brief #181. Cancer Drug Discovery & Development.

[4] Gaieski D, Edwards J, Kallan MJ, Carr BG (2013). Benchmarking the incidence and mortality of severe sepsis in the United States. Crit Care Med.

[5] Zea-Vera A, Ochoa TJ (2015). Challenges in the diagnosis and management of neonatal sepsis. J Trop Pediatr.

[6] Fernando S, Rochwerg B, Seely AJE (2018). Clinical implications of the third international consensus definitions for sepsis and septic shock (Sepsis-3). CMAJ.

[7] Fernando S, Tran A, Taljaard M, Cheng W, Rochwerg B, Seely AJE, Perry JJ (2018). Prognostic accuracy of the quick sequential organ failure assessment for mortality in patients with suspected infection: a systematic review and meta-analysis. Ann Intern Med.

[8] Hiensch R, Poeran J, Saunders-Hao P, Adams V, Powell CA, Glasser A, Mazumdar M, Patel G (2017). Impact of an electronic sepsis initiative on antibiotic use and health care facility-onset clostridium difficile infection rates. Am J Infect Control.

[9] Subbe CP, Kruger M, Rutherford P, Gemmel L (2001). Validation of a modified Early Warning Score in medical admissions. QJM.

[10] Chen Z, Li X, Jin F, Shi Y, Zhang L, Yin H, Zhu Y, Tang X, Lin X, Lu B, Wang Q, Sun L, Zhu X, Qiu L, Xu H, Guo L (2025). Diagnosis of sarcopenia using convolutional neural network models based on muscle ultrasound images: prospective multicenter study. J Med Internet Res.

[11] Rubulotta F, Marshall J, Ramsay G, Nelson D, Levy M, Williams M (2009). Predisposition, insult/infection, response, and organ dysfunction: a new model for staging severe sepsis. Crit Care Med.

[12] Howell M, Talmor D, Schuetz P, Hunziker S, Jones AE, Shapiro NI (2011). Proof of principle: the predisposition, infection, response, organ failure sepsis staging system. Crit Care Med.

[13] Etholén A, Roos T, Hänninen M, Bouri I, Kulmala J, Rahkonen O, Kouvonen A, Lallukka T (2025). Forecasting subjective cognitive decline: AI approach using dynamic Bayesian networks. J Med Internet Res.

[14] Macdonald SPJ, Arendts G, Fatovich DM, Brown SGA (2014). Comparison of PIRO, SOFA, and MEDS scores for predicting mortality in emergency department patients with severe sepsis and septic shock. Acad Emerg Med.

[15] Su L, Xu Z, Chang F, Ma Y, Liu S, Jiang H, Wang H, Li D, Chen H, Zhou X, Hong N, Zhu W, Long Y (2021). Early prediction of mortality, severity, and length of stay in the intensive care unit of sepsis patients based on sepsis 3.0 by machine learning models. Front Med (Lausanne).

[16] Bedoya AD, Futoma J, Clement ME, Corey K, Brajer N, Lin A, Simons MG, Gao M, Nichols M, Balu S, Heller Ka, Sendak M, O'Brien C (2020). Machine learning for early detection of sepsis: an internal and temporal validation study. JAMIA Open.

[17] Fohner AE, Greene JD, Lawson BL, Chen JH, Kipnis P, Escobar GJ, Liu VX (2019). Assessing clinical heterogeneity in sepsis through treatment patterns and machine learning. J Am Med Inform Assoc.

[18] Scott H, Colborn K, Sevick CJ, Bajaj L, Deakyne Davies SJ, Fairclough D, Kissoon N, Kempe A (2021). Development and validation of a model to predict pediatric septic shock using data known 2 hours after hospital arrival. Pediatr Crit Care Med.

[19] Robnik-Šikonja M, Štrumbelj E, Kononenko I (2013). Efficiently explaining the predictions of a probabilistic radial basis function classification network. IDA.

[20] Ribeiro MT, Singh S, Guestrin C (2016). 'Why should I trust you?' explaining the predictions of any classifier. https://doi.org/10.1145/2939672.2939778.

[21] Johnson A, Pollard T, Shen L, Lehman LWH, Feng M, Ghassemi M, Moody B, Szolovits P, Celi LA, Mark RG (2016). MIMIC-III, a freely accessible critical care database. Sci Data.

[22] Goldberger AL, Amaral LA, Glass L, Hausdorff JM, Ivanov PC, Mark RG, Mietus JE, Moody GB, Peng CK, Stanley HE (2000). PhysioBank, PhysioToolkit, and PhysioNet: components of a new research resource for complex physiologic signals. Circulation.

[23] Preechasuk L, Suwansaksri N, Ipichart N, Vannasaeng S, Permpikul C, Sriwijitkamol A (2017). Hyperglycemia and glycemic variability are associated with the severity of sepsis in nondiabetic subjects. J Crit Care.

[24] Magee F, Bailey M, Pilcher DV, Mårtensson J, Bellomo R (2018). Early glycemia and mortality in critically ill septic patients: interaction with insulin-treated diabetes. J Crit Care.

[25] Evans L, Rhodes A, Alhazzani W, Antonelli M, Coopersmith Craig M, French Craig, Machado Flávia R, Mcintyre Lauralyn, Ostermann Marlies, Prescott Hallie C, Schorr Christa, Simpson Steven, Wiersinga W Joost, Alshamsi Fayez, Angus Derek C, Arabi Yaseen, Azevedo Luciano, Beale Richard, Beilman Gregory, Belley-Cote Emilie, Burry Lisa, Cecconi Maurizio, Centofanti John, Coz Yataco Angel, De Waele Jan, Dellinger R Phillip, Doi Kent, Du Bin, Estenssoro Elisa, Ferrer Ricard, Gomersall Charles, Hodgson Carol, Hylander Møller Morten, Iwashyna Theodore, Jacob Shevin, Kleinpell Ruth, Klompas Michael, Koh Younsuck, Kumar Anand, Kwizera Arthur, Lobo Suzana, Masur Henry, McGloughlin Steven, Mehta Sangeeta, Mehta Yatin, Mer Mervyn, Nunnally Mark, Oczkowski Simon, Osborn Tiffany, Papathanassoglou Elizabeth, Perner Anders, Puskarich Michael, Roberts Jason, Schweickert William, Seckel Maureen, Sevransky Jonathan, Sprung Charles L, Welte Tobias, Zimmerman Janice, Levy Mitchell (2021). Surviving sepsis campaign: international guidelines for management of sepsis and septic shock 2021. Crit Care Med.

[26] Tang Y, Sorenson J, Lanspa M, Grissom CK, Mathews V, Brown SM (2017). Systolic blood pressure variability in patients with early severe sepsis or septic shock: a prospective cohort study. BMC Anesthesiol.

[27] Huston JM, Tracey KJ (2011). The pulse of inflammation: heart rate variability, the cholinergic anti-inflammatory pathway and implications for therapy. J Intern Med.

[28] Goldberger AL, Amaral LAN, Hausdorff JM, Ivanov PC, Peng C, Stanley HE (2002). Fractal dynamics in physiology: alterations with disease and aging. Proc Natl Acad Sci U S A.

[29] Bone RC, Balk RA, Cerra FB, Dellinger RP, Fein AM, Knaus WA, Schein RM, Sibbald WJ (1992). Definitions for sepsis and organ failure and guidelines for the use of innovative therapies in sepsis. The ACCP/SCCM Consensus Conference Committee. American College of Chest Physicians/Society of Critical Care Medicine. Chest.

[30] Ganeshan K, Nikkanen J, Man K, Leong YA, Sogawa Y, Maschek JA, Van Ry T, Chagwedera DN, Cox JE, Chawla A (2019). Energetic trade-offs and hypometabolic states promote disease tolerance. Cell.

[31] Walter EJ, Hanna-Jumma S, Carraretto M, Forni L (2016). The pathophysiological basis and consequences of fever. Crit Care.

[32] Thomas-Rüddel DO, Hoffmann P, Schwarzkopf D, Scheer C, Bach F, Komann M, Gerlach H, Weiss M, Lindner M, Rüddel H, Simon P, Kuhn S, Wetzker R, Bauer M, Reinhart K, Bloos F, MEDUSA study group (2021). Fever and hypothermia represent two populations of sepsis patients and are associated with outside temperature. Crit Care.

[33] Ye Y, Li F, Yang B (2024). Exploring the optimal range of pulse oxygen saturation in patients with sepsis: a retrospective study based on MIMIC- IV dataJ. Zhonghua Wei Zhong Bing Ji Jiu Yi Xue.

[34] Pope JV, Jones AE, Gaieski DF, Arnold RC, Trzeciak S, Shapiro NI, Emergency Medicine Shock Research Network (EMShockNet) Investigators (2010). Multicenter study of central venous oxygen saturation (ScvO(2)) as a predictor of mortality in patients with sepsis. Ann Emerg Med.

[35] Sepsis Risk Prediction System. Streamlit Community Cloud.

[36] Wang G, Bennamoun H, Kwok WH, Quimbayo JPO, Kelly B, Ratajczak T, Marriott R, Walker R, Kotz J (2025). Investigating protective and risk factors and predictive insights for aboriginal perinatal mental health: explainable artificial intelligence approach. J Med Internet Res.

